# Desipramine treatment has minimal effects on the brain accumulation of glucocorticoids in P-gp-deficient and wild-type mice

**DOI:** 10.1016/j.psyneuen.2011.03.008

**Published:** 2011-10

**Authors:** Brittany L. Mason, Sarah A. Thomas, Stafford L. Lightman, Carmine M. Pariante

**Affiliations:** a Institute of Pharmaceutical Science, King's College London, London, UK; b Section of Perinatal Psychiatry and Laboratory of Stress, Psychiatry and Immunology (SPI-Lab), Institute of Psychiatry, King's College London, London, UK; c Henry Wellcome Laboratories for Integrative Neuroscience and Endocrinology, University of Bristol, Bristol, UK

**Keywords:** Blood–brain barrier, Glucocorticoids, Desipramine, Antidepressants, Hypothalamic-pituitary-adrenal (HPA) axis

## Abstract

Hyperactivity of the hypothalamic-pituitary-adrenal (HPA) axis in patients with depression can be reduced by antidepressants, which are thought to improve endogenous glucocorticoid-mediated negative feedback. A proportion of peripherally released glucocorticoids need to enter brain tissue, protected by the blood–brain barrier (BBB), in order to achieve this negative feedback effect at the level of the central nervous systems (CNS). The multidrug resistance transporter P-glycoprotein (P-gp) has been shown to actively transport glucocorticoid hormones and has been implicated in the regulation of glucocorticoid access to the CNS. Using an *in situ* brain/choroid plexus perfusion method, we tested the hypothesis that the antidepressant desipramine increases glucocorticoid accumulation in the mouse brain by inhibiting P-gp, following either chronic treatment (8 days, 20 mg/kg/day, IP) or acute administration (20 min brain perfusion in the presence of either 0.9 μM or 10 μM desipramine). Contrary to our hypothesis, chronic treatment with desipramine did not affect the accumulation of [^3^H]dexamethasone in any sample compared to saline-treated mice. Acute desipramine had limited and variable effects on glucocorticoid accumulation in the CNS, with accumulation of [^3^H]dexamethasone increased in the cerebellum, accumulation of [^3^H]cortisol reduced in the frontal cortex, hypothalamus, and cerebellum, and accumulation of [^3^H]corticosterone (the endogenous glucocorticoid in rodents) not affected. Overall, under the conditions tested, these results do not support the hypothesis that treatment with desipramine can inhibit P-gp at the BBB and subsequently increase the accumulation of glucocorticoids in the brain.

## 1 Introduction

A disruption of the hypothalamic-pituitary-adrenal (HPA) axis, the hormonal system that regulates the body's response to stress, has been consistently found in a number of psychiatric disorders (Pariante, 2009). Due to inputs from both limbic circuits and the brain stem, the HPA axis can respond to both psychological and physical stressors (Lightman, 2008). Stimulation by a stressor induces the paraventricular nucleus of the hypothalamus (PVN) to release corticotropin-releasing hormone (CRH), signaling the anterior pituitary to release adrenocorticotropin hormone (ACTH), which in turn signals the release of glucocorticoids from the adrenal cortex, in the form of cortisol in humans and corticosterone in rodents (Lightman, 2008). Glucocorticoids then bind to intracellular nuclear receptors, the mineralocorticoid receptor (MR) and the glucocorticoid receptor (GR), in a number of target tissues (reviewed in Pariante and Miller, 2001).

Hyperactivity of the HPA axis has been found in a large percentage of patients with depression and, though the exact site of the disruption in HPA axis regulation in patients with depression is unknown, a key implicated factor is the glucocorticoid-mediated negative feedback by which endogenous glucocorticoids control HPA axis activity (Pariante and Lightman, 2008). Increased concentrations of plasma and cerebrospinal fluid (CSF) cortisol, non-suppression of cortisol secretion following administration of the synthetic glucocorticoid dexamethasone (in the dexamethasone suppression or the dexamethasone/CRH test), and increased volumes of the pituitary and adrenal glands (Pariante, 2009), all suggest that cortisol is hypersecreted and that negative feedback on the HPA axis is reduced. Additionally, it has been shown that normalization of these HPA abnormalities is necessary before any resolution of clinical symptoms (Holsboer et al., 1982); indeed, recent studies suggested that HPA hyperactivity may be an indicator of a biological vulnerability to depression, as shown by persistency of HPA axis abnormalities in both acutely ill and recovered depressed patients (Vreeburg et al., 2009; Juruena et al., 2010). Much work has examined the role of GR in this HPA hyperactivity and the impaired glucocorticoid-mediated negative feedback, as the GR seems to be more important when endogenous hormones are high, and has found that GR function in patients with depression is impaired (Pariante, 2009). Interestingly, antidepressants have been shown to exert direct effects on the GR (and on the MR), including increasing receptor expression and function, which are likely to improve negative feedback on the HPA axis and thus decreasing basal and stress-induced glucocorticoid secretion in rodents and humans (Pariante, 2009).

In order to mediate the negative feedback control on the HPA axis, a proportion of these glucocorticoids must enter brain tissue, which is protected by the blood–brain barrier (BBB), found at the level of the cerebral capillary endothelial cells. In addition to the BBB, the free movement of molecules from the blood is also restricted by the blood–cerebrospinal fluid (CSF) barrier (BCSFB) which is found at the fluid-secreting epithelium of the choroid plexus and at the arachnoid membrane (Gazzin et al., 2008; Johanson et al., 2008). Fenestrated capillaries that feed the choroid plexus allow for relatively free movement of molecules into choroid plexus tissue, with some of these molecules then being transported into CSF. The ventricular distribution of CSF-borne signals occurs by bulk flow and there is no physical restriction in the movement of molecules from the CSF to the brain; however, movement from CSF to brain is limited (Johanson et al., 2008). Nevertheless, passage through the BCSFB may provide an “easier” route of access to the brain compared with access through the restrictive BBB. Of special note is the differential expression of transporters in capillary endothelium (BBB) and choroidal epithelium (BCSFB) that may contribute to differential access of the same molecules to each barrier compartment (Gazzin et al., 2008). Additionally, there are central nervous system (CNS) regions that have non-barrier type capillaries; for example, fenestrated capillaries feed the posterior pituitary gland allowing free exchange of blood-borne molecules between blood and tissue. The posterior pituitary gland is an example of a circumventricular organ (CVO), which are a group of self-contained structures surrounded by physical restrictions, preventing the free movement of molecules into non-CVO tissues (Johnson and Gross, 1993). Thus, as CNS tissues have a varied ease of molecule penetration from the peripheral circulation, it is necessary to evaluate the access to different tissues with reference to the physiological penetration across the respective barriers.

The multidrug resistance transporter P-glycoprotein (P-gp) has been characterized as a major contributor in the phenomenon of highly lipophilic molecules penetrating the brain from the blood at a much lower rate than expected, and has been found to have numerous substrates, inhibitors and modulators (Schinkel, 1999). In humans, MDR1 codes for the major drug-transporting isoform of P-gp (Schinkel, 1999) and the drug-transporting isoforms in rodents are mdr1a and mdr1b, which together share 80% homology with the human MDR1 (Borst and Schinkel, 1996). P-gp is expressed in a number of tissues, including the liver, kidneys, intestine, and adrenal cortex (Schinkel et al., 1997). Most importantly, P-gp is expressed at the blood–CNS barriers; specifically, P-gp has been identified in human and murine cerebral capillary endothelial cells and found to actively transport substrates from the basolateral (brain) side to the luminal (blood) side of the cell (Cordon-Cardo et al., 1989; Schinkel, 1999). It has also been detected in rat and rabbit choroid plexus (Choudhuri et al., 2003; Kassem et al., 2007), as well as in isolated choroid plexus lysates from wild-type FVB-abcb1a/b(+/+) mice, but not those from the specific P-gp knockout mouse strain, FVB-abcb1a/b(−/−) (Rao et al., 1999). Moreover, P-gp has been shown to actively transport glucocorticoid hormones and has been implicated in the regulation of glucocorticoid access to the CNS (Meijer et al., 1998; Karssen et al., 2001; Uhr et al., 2002), although perhaps not to such a large extent as originally hypothesized (Mason et al., 2008).

In 2004, in this Journal, we hypothesized that the activity of P-gp at the BBB may be a target of antidepressant action (Pariante et al., 2004). Specifically, we proposed that antidepressants can inhibit P-gp at the BBB, thus increasing the access of glucocorticoids to the brain and improving the glucocorticoid-mediated negative feedback. This hypothesis was based on our *in vitro* data, showing that antidepressants increased GR activity not only directly, by activating the translocation of the GR from the cytoplasm to the nucleus, but also indirectly, by increasing intracellular concentration of glucocorticoids via inhibition of P-gp (Pariante et al., 1997, 2001). Specifically, we found that intracellular levels of [^3^H]cortisol, in mouse fibroblasts LMCAT cells and in primary rat neuronal cultures, were increased by 24 h incubation of cells with the P-gp inhibitor, verapamil, as well as by the antidepressants, clomipramine and fluoxetine (Pariante et al., 2003a,b); these effects were abolished when the antidepressants and verapamil were incubated together (Pariante et al., 2003a,b). Our later animal data also showed that the effects of antidepressant treatment on GR expression and function were different in mice deficient for P-gp (Yau et al., 2007). Specifically, 1 week of chronic desipramine treatment increased GR mRNA transcription in the hippocampus and reduced plasma corticosterone concentrations of FVB-abcb1a/b(+/+) mice, but not in FVB-abcb1a/b(−/−) mice, which led us to conclude that P-gp is necessary for desipramine to induce the effects on the GR expression and HPA axis function in mice. However, until today we had not yet tested whether antidepressant treatment could specifically increase glucocorticoid entry into the brains of mice by inhibiting P-gp.

The aim of this current study is to test the hypothesis that antidepressant treatment directly increases glucocorticoid accumulation in the brain by a P-gp-mediated mechanism. Our initial work in this area demonstrated that the accumulation of [^3^H]dexamethasone is greater in the brains of FVB-abcb1a/b(−/−) mice compared to FVB-abcb1a/b(+/+) mice (Mason et al., 2008) and, in order to address our hypothesis, we have now used our *in situ* brain/choroid plexus perfusion in these mice to examine the effect of chronic treatment (8 days) with desipramine on the brain accumulation of [^3^H]dexamethasone. We have also conducted cross-competition studies to determine whether acute inhibition of P-gp by desipramine (20 min) can increase the brain accumulation of [^3^H]dexamethasone, as well as of the endogenous [^3^H]glucocorticoids, cortisol and corticosterone.

## 2 Method

All procedures are within the guidelines of the Animals (Scientific Procedures) Act, 1986, UK.

### 2.1 Animals

Adult FVB-abcb1a/b (+/+) and FVB-abcb1a/b(−/−) mice were imported from Taconic Farms Inc. (Germantown, NY, USA) and a breeding colony was established at King's College London under an academic breeding agreement. Genotype was confirmed by PCR analysis (Harlan UK Ltd., Hillcrest, Belton, Loughborough, UK) and it is recognized that Dr. Alfred Schinkel of the Netherlands Cancer Institute is the creator of the abcb1a/b-deficient mice. All animals were maintained under standard conditions of temperature and lighting (lights on at 0700 h and off at 1900 h) and given food and water *ad libitum*.

### 2.2 Materials

#### 2.2.1 Radiolabeled substances

[^3^H]cortisol (74.0 Ci/mmol), [^3^H]corticosterone (79.0 Ci/mmol), [^3^H]dexamethasone (89.0 Ci/mmol) were purchased from GE Healthcare (Buckinghamshire, UK). [^14^C]sucrose (0.49 Ci/mmol) was purchased from Moravek Biochemicals (Brea, CA, USA).

#### 2.2.2 Unlabeled substances

Desipramine hydrochloride was purchased from Sigma (Poole, Dorset, UK). All materials were stored as recommended by the suppliers.

### 2.3 Procedures

#### 2.3.1 *In situ* brain/choroid plexus perfusion technique

This technique has been described and validated previously (Sanderson et al., 2007). Adult male mice (25–40 weeks and 25–46 g) were anaesthetized with a medetomidine hydrochloride (2 mg/kg, IP) and ketamine hydrochloride solution (150 mg/kg, IP) and heparinized (100 Units, IP). The body cavity was opened and the left ventricle cannulated with a fine needle (25 gauge) connected to a perfusion circuit. A Watson–Marlow peristaltic pump (323S/RL, Cornwall, UK) was used to perfuse the heart *in situ* with a warmed (37 °C) and oxygenated (95% O_2_; 5% CO_2_) artificial plasma (modified Krebs-Henseleit mammalian Ringer solution) as detailed previously (Mason et al., 2008). With the start of perfusion (5.0 ml/min), the right atrium was sectioned to create an open circuit and allow drainage of the artificial plasma. In all experiments, a 2.5 min pre-drug perfusion of artificial plasma ensured removal of endogenous glucocorticoids from the brain vasculature. [^3^H]dexamethasone (3.9 nM), [^3^H]cortisol (3.6 nM) or [^3^H]corticosterone (3.8 nM) along with [^14^C]sucrose (vascular or extracellular space marker; 0.5–1.0 nM), was then administered by a slow-drive syringe pump (model 22; Harvard Apparatus; Kent, UK) into the artificial plasma. Following the desired isotope perfusion time period up to 20 min, the mouse was decapitated. Selected areas of the CNS, both HPA axis-related regions and major brain regions (frontal cortex, hippocampus, hypothalamus, cerebellum, fourth ventricle choroid plexus, and pituitary gland, consisting of both anterior and posterior sections), were dissected and weighed. All these samples, together with 100 μl artificial plasma samples, were prepared for liquid scintillation counting as described below.

#### 2.3.2 Antidepressant treatment and corticosterone measurement

Desipramine hydrochloride (dissolved in 0.9% saline at 1 mg/ml) was injected (10 mg/kg, IP) twice daily for 8 days. Injections were given between 0900–0930 h and 1600–1630 h to coincide with the peak and trough of the natural corticosterone fluctuation (Kotelevtsev et al., 1997). Before the perfusion, animals were subjected to blood sampling from the saphenous leg vein, and then deeply anesthetized for the 5 min *in situ* brain/choroid plexus perfusion, as detailed above. Blood samples were centrifuged at 13,793 × *g* for 5 min at 20 °C and the resulting plasma sample was snap frozen on dry ice. The plasma samples were transported to Dr. J.L.W. Yau (University of Edinburgh) and were there analyzed for the concentration of corticosterone present, as described in Yau et al. (2007).

It is of note that this study design is different from that in our previous paper by Yau et al. (2007), in which all animals were killed by decapitation, in the morning, 16 h after the last injection. The perfusion procedure must be conducted immediately after anesthetizing of the animals; hence the sacrificing of the mice was staggered throughout the day (0900–1600 h). All mice were perfused the day following the last desipramine injection, and thus were subjected to the brain/choroid plexus perfusion 17–24 h after last injection.

#### 2.3.3 Cross-competition studies

In separate sets of single-time point experiments, the uptake of [^3^H]dexamethasone (3.9 nM), [^3^H]cortisol (3.6 nM), or [^3^H]corticosterone (3.8 nM) was determined in the presence of unlabeled desipramine (0.9 μM and10 μM, dissolved in the artificial plasma) and compared to perfusions in which no unlabeled desipramine was present. 0.9 μM corresponds to the upper limit of the therapeutic plasma concentration in patients taking desipramine (Yau et al., 2007) and 10 μM has previously been shown to induce GR translocation to the nucleus *in vitro* (Pariante et al., 1997). All perfusions (including wild-type FVB-abcb1a/b(+/+) controls, i.e., no desipramine present in the artificial plasma) were 20 min in length, in order to allow sufficient time for the desipramine to inhibit all potential transporters; desipramine was present in the artificial plasma solution from the beginning of the perfusion time. In all perfusion experiments, [^14^C]sucrose (0.5–1.0 nM) was present as a vascular or extracellular space marker.

#### 2.3.4 Liquid scintillation counting

All samples were solubilized over at least 48 h in 0.5 ml of Solvable (PerkinElmer Life and Analytical Sciences, Boston, MA, USA). All samples were then vortexed, 3.5 ml of scintillation counting fluid (Lumasafe; PerkinElmer Life and Analytical Sciences) was then added and the samples vortexed again. The samples were then placed in a Packard TriCarb 2100 or 2900TR (PerkinElmer, Beaconsfield, UK) liquid scintillation counter for estimation of [^3^H] and [^14^C] radioactivities. All results were corrected for background radioactivity.

#### 2.3.5 Expression of results

Data from all experiments are presented as mean ± standard error of the mean. The concentration of [^3^H] or [^14^C] radioactivity in the brain tissue (dpm g^−1^) is expressed as a percentage of that in artificial plasma (dpm ml^−1^) and is referred to as *R*_Tissue_ %. In order to account for any differences in vascular space, in reference to brain regions protected by the BBB, or extracellular space, in reference to CNS samples that are not protected by the BBB, the *R*_Tissue_% values of the radioactive glucocorticoids were corrected with the *R*_Tissue_ values of [^14^C]sucrose, and termed *R*_CorrTissue_% and are referred to as “vascular space-“, or “extracellular space-corrected”, respectively, and are used when comparing glucocorticoid values between strains.

#### 2.3.6 Data analysis

The data from all the experiments are presented as mean ± standard error of the mean. The GraphPad statistical program (GraphPad Prism, 5.0b Mac Version) was used for all determinations. The level of significance was set at (*P* < 0.05). Two-Way ANOVAs were used to compare uptake of [^3^H]dexamethasone between treatment groups for each individual region following chronic desipramine treatment; Student's *t*-tests were used to compare plasma corticosterone concentrations between groups; One-Way ANOVAs, followed by Tukey's HSD post hoc tests, were used to compare uptake of [^3^H]dexamethasone, [^3^H]cortisol, or [^3^H]corticosterone between groups for each individual region in the cross-competition studies.

## 3 Results

### 3.1 [^3^H]dexamethasone perfusion following chronic desipramine treatment

#### 3.1.1 Brain regions

**Figure 1:**
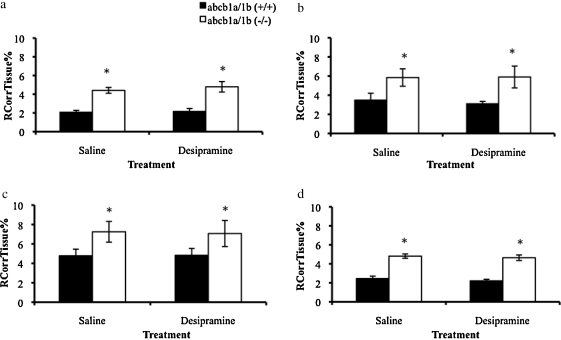
*R*_CorrTissue_% (ml 100 g^−1^) of [^3^H]dexamethasone in the brain regions (frontal cortex, 1a; hippocampus, 1b; hypothalamus, 1c; cerebellum, 1d) following 5 min perfusion in saline-treated and desipramine-treated abcb1a/b(+/+) and abcb1a/b(−/−) mice (abcb1a/b(+/+) mice: *n* = 12; abcb1a/b(−/−) mice: *n* = 11). * Denotes significance compared with FVB-abcb1a/b(+/+) mice (*P* < 0.05, Two-Way ANOVAs, vascular space-corrected).

The vascular space-corrected [^3^H]dexamethasone values are presented in Fig. 1. The accumulation of [^3^H]dexamethasone was significantly greater in the frontal cortex, hippocampus, hypothalamus, and cerebellum of FVB-abcb1a/b(−/−) mice compared to FVB-abcb1a/b(+/+) mice (*P* < 0.0001, *P* = 0.018, *P* = 0.034, *P* < 0.0001, respectively, Two-Way ANOVAs, vascular space-corrected). Chronic treatment with desipramine did not significantly affect the accumulation of [^3^H]dexamethasone in any brain region studied when compared to saline-treated controls, nor were there any interactions between factors (*P* > 0.05, Two-Way ANOVAs, vascular space-corrected).

#### 3.1.2 Choroid plexus and pituitary gland

**Figure 2:**
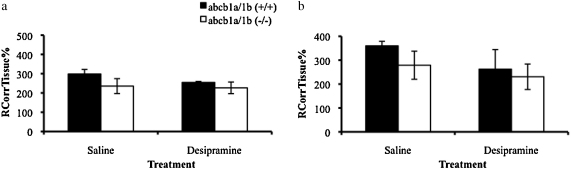
*R*_CorrTissue_% (ml 100 g^−1^) of [^3^H]dexamethasone in the choroid plexus (2a) and pituitary gland (2b) following 5 min perfusion in saline-treated and desipramine-treated abcb1a/b(+/+) and abcb1a/b(−/−) mice (abcb1a/b(+/+) mice: *n* = 12; abcb1a/b(−/−) mice: *n* = 11).

The extracellular space-corrected [^3^H]dexamethasone values are presented in Fig. 2. The accumulation of [^3^H]dexamethasone was not significantly different between strains and chronic treatment with desipramine did not significantly affect the uptake of [^3^H]dexamethasone in either the choroid plexus or in the pituitary gland (*P* > 0.05, Two-Way ANOVAs, corrected for extracellular space).

#### 3.1.3 Plasma corticosterone

**Figure 3:**
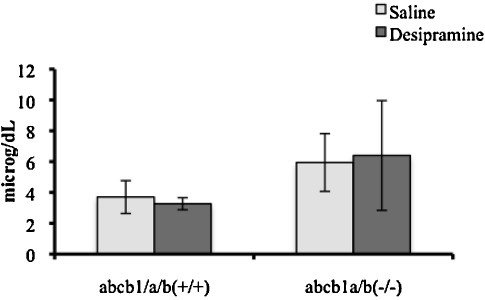
Plasma corticosterone (μg/dL) levels in saline-treated or desipramine-treated abcb1a/b(+/+) and abcb1a/b(−/−) mice (abcb1a/b(+/+) mice: *n* = 10; abcb1a/b(−/−) mice: *n* = 8). Desipramine was injected (10 mg/kg, IP) twice daily for 8 days.

There was no statistically significant difference in the concentration of plasma corticosterone (μg/dL) following chronic treatment with desipramine when all samples were analyzed together (*P* > 0.05, Student's *t*-tests, Fig. 3). However, it is important to emphasize that mice were sacrificed at different times of the day and at varying intervals after the last injection (i.e., 17–24 h; see Section 2). Of note, when we analyzed the corticosterone concentrations only in those animals sacrificed in the morning (*n* = 9; 17–20 h after the last injection), corticosterone concentrations were lower following desipramine (by 20%) but this did not attain statistical significance (*P* = 0.7, Student's *t*-test, data not shown).

### 3.2 [^3^H]glucocorticoid accumulation with acute desipramine exposure

In order to further investigate the effect of desipramine treatment on the CNS accumulation of [^3^H]glucocorticoids, we have conducted acute studies using only a 20 min desipramine exposure period In FVB-abcb1a/b(+/+) mice (i.e., wild-type controls). This allowed us to investigate the interaction of desipramine with any possible transporters.

#### 3.2.1 Brain regions

**Figure 4:**
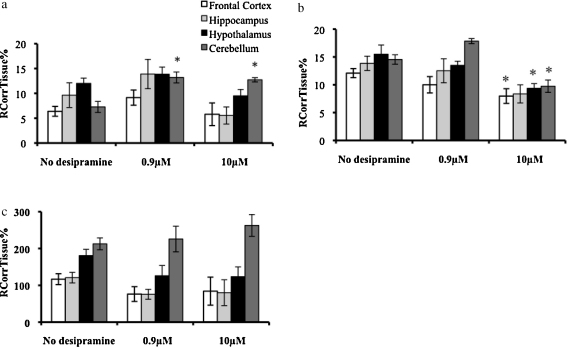
*R*_CorrTissue_% (ml 100 g^−1^) of [^3^H]dexamethasone (4a), [^3^H]cortisol (4b), or [^3^H]corticosterone (4c) in the brain regions following 20 min perfusion in the absence and presence 0.9 and 10 mM) of unlabeled desipramine in mice (*n* = 12 for [^3^H]dexamethasone; *n* = 14-19 for [^3^H]cortisol; *n* = 17–28 for [^3^H]corticosterone) * Denotes significance compared with wild-type control mice (*P* < 0.05, Tukey's HSD, following One-Way ANOVA).

The vascular space-corrected [^3^H]glucocorticoid values are presented in Fig. 4. The accumulation of [^3^H]dexamethasone in the cerebellum was significantly increased in the presence of both concentrations of unlabeled desipramine (*P* = 0.0052, One-Way ANOVA, vascular-space corrected, *P* < 0.01 for 0.9 μM and *P* < 0.05 for 10 μM, Tukey's HSD) compared to wild-type control mice. There were no changes in [^3^H]dexamethasone accumulation observed in the frontal cortex, hippocampus or hypothalamus in the presence of either concentration of desipramine.

The accumulation of [^3^H]cortisol in the frontal cortex, hypothalamus, and cerebellum was significantly decreased in the presence of 10 μM of unlabeled desipramine compared to wild-type control mice (*P* = 0.0425, *P* = 0.0205, *P* = 0.0004, respectively, Tukey's HSD following One-Way ANOVAs, vascular-space corrected). There was also a decrease in the accumulation of [^3^H]cortisol in the hippocampus when 10 μM was present; however, this did not attain statistical significance (*P* = 0.07, Tukey's HSD following One-Way ANOVA, vascular space-corrected). No effect on [^3^H]cortisol accumulation was not observed in the presence of 0.9 μM of desipramine.

The accumulation of [^3^H]corticosterone was not significantly different in any brain regions, in the presence of either concentration of unlabeled desipramine (*P* > 0.05, One-Way ANOVAs, vascular space-corrected).

#### 3.2.2 Choroid plexus and pituitary gland

**Figure 5:**
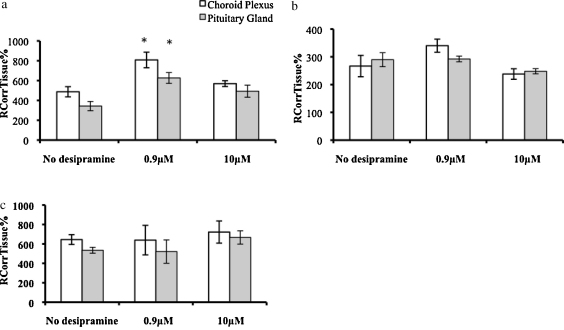
*R*_CorrTissue_% (ml 100 g^−1^) of [^3^H]dexamethasone (5a), [^3^H]cortisol (5b), or [^3^H]corticosterone (5a) in the choroid plexus or pituitary gland following 20 min perfusion in the presence of 0.9 mM or 10 mM unlabeled desipramine in FVB mice (*n* = 12 for ^3^H]dexamethasone; *n* = 19 for [^3^H]cortisol; *n* = 26 for [^3^H]corticosterone); * Denotes significance compared with wild-type control mice (*P* < 0.05, Tukey's HSD, following One-Way ANOVA).

The extracellular space-corrected [^3^H]glucocorticoid values are presented in Fig. 5. The accumulation of [^3^H]dexamethasone in the choroid plexus and in the pituitary gland was significantly increased in the presence of 0.9 μM of unlabeled desipramine compared to wild-type controls (*P* < 0.05, *P* < 0.01, respectively, Tukey's HSD following One-Way ANOVAs, corrected for extracellular space). There was no effect of the 10 μM concentration of desipramine on [^3^H]dexamethasone accumulation in the choroid plexus or pituitary gland. The accumulations of [^3^H]cortisol and of [^3^H]corticosterone were not significantly different in the absence or presence of either concentration of unlabeled desipramine in either the choroid plexus or pituitary gland (*P* > 0.05, One-Way ANOVAs, corrected for extracellular space).

## 4 Discussion

These experiments were conducted in order to directly test the hypothesis that antidepressant treatment in mice can inhibit P-gp and subsequently increase the accumulation of glucocorticoids in the brain (Pariante et al., 2004). In our previously published multiple-time point perfusion experiments in mice (Mason et al., 2008), the most robust and consistent effects of the efflux of glucocorticoids by P-gp were detected with [^3^H]dexamethasone, rather than [^3^H]cortisol or [^3^H]corticosterone, thus the effect of chronic desipramine treatment was examined using [^3^H]dexamethasone. Consistent with our previously published data (Mason et al., 2008), the accumulation of [^3^H]dexamethasone was significantly greater in the brain regions (frontal cortex, hippocampus, hypothalamus, and cerebellum) of FVB-abcb1a/b(−/−) mice compared with FVB-abcb1a/b(+/+) mice. Contrary to our hypothesis, treatment for 8 days with desipramine did not significantly affect the accumulation of [^3^H]dexamethasone in any sample compared to saline-treated mice.

As our chronic antidepressant treatment experiment did not indicate that desipramine was able to modulate the function of P-gp, we used cross-competition studies to test if acute administration of desipramine was able to directly inhibit P-gp and increase the CNS accumulation of [^3^H]dexamethasone, [^3^H]cortisol, or [^3^H]corticosterone. We chose a longer perfusion time of 20 min for these experiments in order to allow time for the desipramine in the artificial plasma to fully inhibit any possible transport mechanisms. We used both 0.9 μM, which corresponds to the upper limit of the therapeutic plasma concentration found in patients taking desipramine (Yau et al., 2007), and 10 μM, which has previously been shown (for desipramine and other antidepressants) to increase GR function and intracellular concentration of glucocorticoids *in vitro* (Pariante et al., 1997, 2001, 2003a,b). The cross-competition studies are designed in such a way as to inhibit the major contributing transporters and to indicate directionality, i.e., a decrease in [^3^H]glucocorticoid in the presence of an excess of unlabeled desipramine indicates the inhibition of an influx transporter. We found that acute desipramine had some effects on glucocorticoid entry into the brain, but overall these effects suggested only a variable and limited action of desipramine on P-gp, which was not consistent across the glucocorticoids tested. Interestingly, we found evidence of the saturable transport of [^3^H]dexamethasone and of [^3^H]cortisol, but no evidence of the saturable transport of [^3^H]corticosterone. Specifically, the accumulation of [^3^H]dexamethasone in the cerebellum was increased in the presence of both 0.9 μM and10 μM of unlabeled desipramine, indicating the presence of an efflux mechanism able to transport [^3^H]dexamethasone which is inhibited by desipramine. As we previously identified increased accumulation of [^3^H]dexamethasone in this region in FVB-abcb1a/b(−/−) mice compared to FVB-abcb1a/b(+/+) mice (Mason et al., 2008), the transporter identified in this current study may indeed be P-gp. These results are consistent with our *in vitro* data and with our original hypothesis, although clearly this effect is not present in other brain areas or indeed after chronic desipramine administration. The accumulation of [^3^H]dexamethasone in the choroid plexus and in the pituitary gland was also increased in the presence of 0.9 μM of unlabeled desipramine, but this is unlikely to be due to P-gp inhibition as this transporter is not present in the pituitary (Mason et al., 2008). In contrast to the data for [^3^H]dexamethasone, the accumulation of [^3^H]cortisol in the frontal cortex, hypothalamus, and cerebellum (and, not significantly, in the hippocampus), was reduced in the presence of 10 μM of unlabeled desipramine, suggesting that there may be an influx transporter present at the level of the BBB which is sensitive to desipramine. It is possible that this is the transporter oatp2, previously identified in the cerebellum (Mason et al., 2010). Finally, and perhaps most importantly in this context, the accumulation of [^3^H]corticosterone, the endogenous glucocorticoid in mice, was not significantly different in the brain regions in the presence of either concentration of unlabeled desipramine, and thus, under the conditions tested, our findings do not support the notion that antidepressants modulate the HPA axis in these animals by direct interactions with transporters at the BBB which transport glucocorticoids.

A concern with our current findings arises when comparing these present data to our previous findings in mice using virtually the same antidepressant regime of 20 mg/day of desipramine for 7 days (Yau et al., 2007). In this previous study, we found that desipramine decreases corticosterone concentrations in both FVB/N controls and in FVB-abcb1a/b(−/−) mice, whereas in the current study we have not found an effect of chronic desipramine treatment on plasma corticosterone concentrations. We did however have significant study design differences; specifically, in our previous paper, blood was collected when all animals were killed by decapitation in the morning, 16 h after the last injection, whereas in the current paper, blood was collected through blood sampling from the saphenous leg vein just before the anesthesia, and mice were sacrificed at different times of the day and following different intervals ranging from 17 to 24 h after the last injection. Clearly, the conditions in the present study, while being optimal for the brain perfusion experiments, are far from ideal to detect an effect of desipramine on corticosterone concentrations, since these could be influenced both by circadian rhythms and by the direct effects of variable concentrations of desipramine. Indeed, in the subsample of mice sacrificed in the morning we did find a small (albeit not significant) reduction of corticosterone levels by desipramine. Therefore, we believe that our different results, showing a lack of effect of desipramine on plasma corticosterone concentrations, shall not be considered a lack of replication of our previous findings but rather a consequence of the differences in study design. An additional limitation of the study is that we collected a small volume of blood from live animals and thus were unable to obtain enough plasma to analyze the desipramine concentration in addition to the corticosterone concentration, nor we were able to source extra brain tissue to analyze for glucocorticoid receptor (GR) mRNA values to compare to our previous data published in Yau et al. (2007), as all brain tissue was submitted to radioactivity counting.

Overall, under the conditions tested, these results do not support our original hypothesis that chronic treatment with antidepressants inhibits P-gp and subsequently increases the accumulation of glucocorticoids in the brain. Of note, since we initially proposed this hypothesis in 2003, support for this null finding has been accumulating. For example, one study in mice has shown that treatment with fluoxetine, amitriptyline, and mirtazapine fail to change the expression of P-gp or to disrupt the linear relationship between corticosterone levels in the plasma and in the brain (Weber et al., 2006). More importantly, two recent studies have evaluated brain P-gp function *in vivo* using positron emission tomography (PET; de Klerk et al., 2009, 2010). In studies in rats, de Klerk et al. (2009) have found that antidepressant treatment increases, rather than inhibits, P-gp function, and Clarke et al. (2009) have shown that administration of the P-gp inhibitor verapamil increases antidepressant concentrations in brain. Moreover, and of particular relevance to our hypothesis, the same authors have also found that P-gp activity is increased, rather than inhibited, in patients with major depression who are taking antidepressants (de Klerk et al., 2009). Therefore, our data indicating that desipramine does not increase [^3^H]dexamethasone in the brains of mice are consistent with the most recent evidence, which further supports us in refuting our original hypothesis.

However, it is important to interpret our present findings also in the light of our previous work which led us to conduct this study. *In vitro*, P-gp is clearly inhibited by several antidepressants, as we and others have demonstrated using well-validated pharmacological assays to measure P-gp activity (Pariante et al., 2003b; Weber et al., 2005). However, in pharmacological assays, desipramine induces only 14% of inhibition of P-gp activity at 10 μM, a concentration that is close to the maximum concentration that can be achieved *in vivo* (Weber et al., 2005). Other antidepressants also show similar degrees of P-gp inhibition (Pariante et al., 2003b; Weber et al., 2005); therefore, it is possible that this small degree of inhibition, while able to increase intracellular glucocorticoids *in vitro*, is not enough to obtain an effect on glucocorticoid entry into the brain *in vivo*, as it certainly does not compare to the P-gp deficiency present in FVB-abcb1a/b(−/−) mice. Moreover, in the original *in vitro* work in LMCAT cells and primary neuronal cortex, P-gp was never fully identified as the transporter in question, and it is known that the inhibitor used, verapamil, can also inhibit the efflux transporter BCRP (Matsson et al., 2009). Certainly, it is intriguing that our previous study in mice has found that virtually the same antidepressant regime used in the present study induces GR upregulation in the brain of FVB/N controls mice but not of FVB-abcb1a/b(−/−), thus supporting the notion that P-gp is involved in the effects of antidepressants on the HPA axis (Yau et al., 2007). These effects, however, could be explained by changes occurring in the periphery rather than at the BBB, since P-gp is present also in the liver, kidneys, intestine, and adrenal cortex (Schinkel, 1999). Taken together with the aforementioned studies by de Klerk et al. (2009, 2010) in rats and humans, and our own recent work finding no evidence of P-gp contribution to entry of endogenous glucocorticoids to the brain in mice (Mason et al., 2008), our present study demonstrates that desipramine is unlikely to regulate glucocorticoid effects on the brain via P-gp inhibition.

Finally, it is very important to stress in this context that a *direct pathway* by which antidepressants activate GR translocation and increase GR function has been clearly demonstrated, via activation of intracellular kinases and phosphorylation of the GR (reviewed in Mason and Pariante, 2006; Carvalho and Pariante, 2008; Anacker et al., 2011a). Of note is our recent paper showing that antidepressants increase human hippocampal neurogenesis via a GR-dependent mechanism that requires PKA-signaling and GR phosphorylation (Anacker et al., 2011b). Moreover, antidepressants can regulate the HPA axis via different mechanisms besides increasing GR binding to DNA. For example, recent data have shown that, in man, glucocorticoids can have very rapid non-linear effects on feedback at the level of the pituitary gland, which comes on within a few minutes and would not need to be associated with any change in intracellular glucocorticoid levels (Russell et al., 2010). Indeed, and in keeping with this concept, it is now clear that there is only episodic exposure of tissues to endogenous cortisol, as most circulating cortisol and corticosterone are inactive due to binding by corticosterone binding globulin (CBG), and thus tissue levels can only rise sharply at the endogenous cortisol peak pulses when the binding to CBG is saturated (Cameron et al., 2010). Therefore, these negative findings do not detract from the overall model that the HPA axis is a *direct target* of antidepressant action. On the contrary, by clarifying mechanisms that are unlikely to be relevant, these data move us forward in exploring alternative molecular pathways by which antidepressants can alter these biological systems.

## Role of funding source

This work was supported by a UK Medical Research Council (MRC) Clinician Scientist Fellowship to C.M. Pariante (G108/603), and was supported by the Wellcome Trust [080268], granted to S.A. Thomas. Neither funding source had any further role in study design; in the collection, analysis and interpretation of data; in the writing of the report; and in the decision to submit the paper for publication.

## Conflict of interest statement

The authors have no conflict of interest to declare.
